# Dedifferentiated Chondrosarcoma: A Report of a Rare and Intriguing Case

**DOI:** 10.7759/cureus.68452

**Published:** 2024-09-02

**Authors:** Sumithra A, Vallal Kani, Sudha Vasudevan, Muthuvel Esakki

**Affiliations:** 1 Department of Pathology, Saveetha Medical College and Hospital, Saveetha Institute of Medical and Technical Sciences, Saveetha University, Chennai, IND

**Keywords:** bone tumor, sarcoma, abrupt transition, dedifferentiated, chondrosarcoma

## Abstract

Dedifferentiated chondrosarcomas (DDCS) are highly aggressive tumors with poor outcomes. Chondrosarcoma (CS) can be categorized based on localization (periosteal, central, and peripheral) or histology, with conventional CS being the most common subtype. However, rarer histological types, such as clear-cell CS, DDCS, and mesenchymal CS, also exist. We present a unique case of DDCS in a 28-year-old male who presented with swelling on the proximal phalanx of the fourth finger. Radiographs showed sclerotic margins and a central diaphyseal lytic lesion. Immunohistochemical analysis using S-100 and Ki67 markers confirmed the diagnosis of DDCS. Treatment involved a multidisciplinary approach, including surgical resection, adjuvant chemotherapy, and radiation therapy. This case underscores the importance of early identification of DDCS and the need for tailored management strategies to address its specific characteristics.

## Introduction

Dedifferentiated chondrosarcoma (DDCS) is an uncommon and highly aggressive variant of CS, accounting for only 1-2% of all primary bone cancers, and is associated with a very poor prognosis. The term DDCS was introduced by Dahlin and Beabout in 1971 [1,2]. It refers to the presence of high-grade non-cartilaginous sarcoma intermixed with low- or intermediate-grade CS [3].

DDCS predominantly affects the appendicular skeleton, with primary tumors commonly located in the femur, humerus, and pelvis. Only four cases of DDCS have been documented in the small bones of the hands and feet [1]. Radiographically, DDCS typically presents as a destructive lesion, often accompanied by a soft tissue tumor. The disease is known for its aggressive nature, with a five-year survival rate ranging from 7% to 24% [4,5].

We report a case of a 28-year-old male with a bone tumor initially suspected to be an enchondroma. However, further examination following a ray amputation revealed it to be DDCS.

## Case presentation

**Figure 1:**
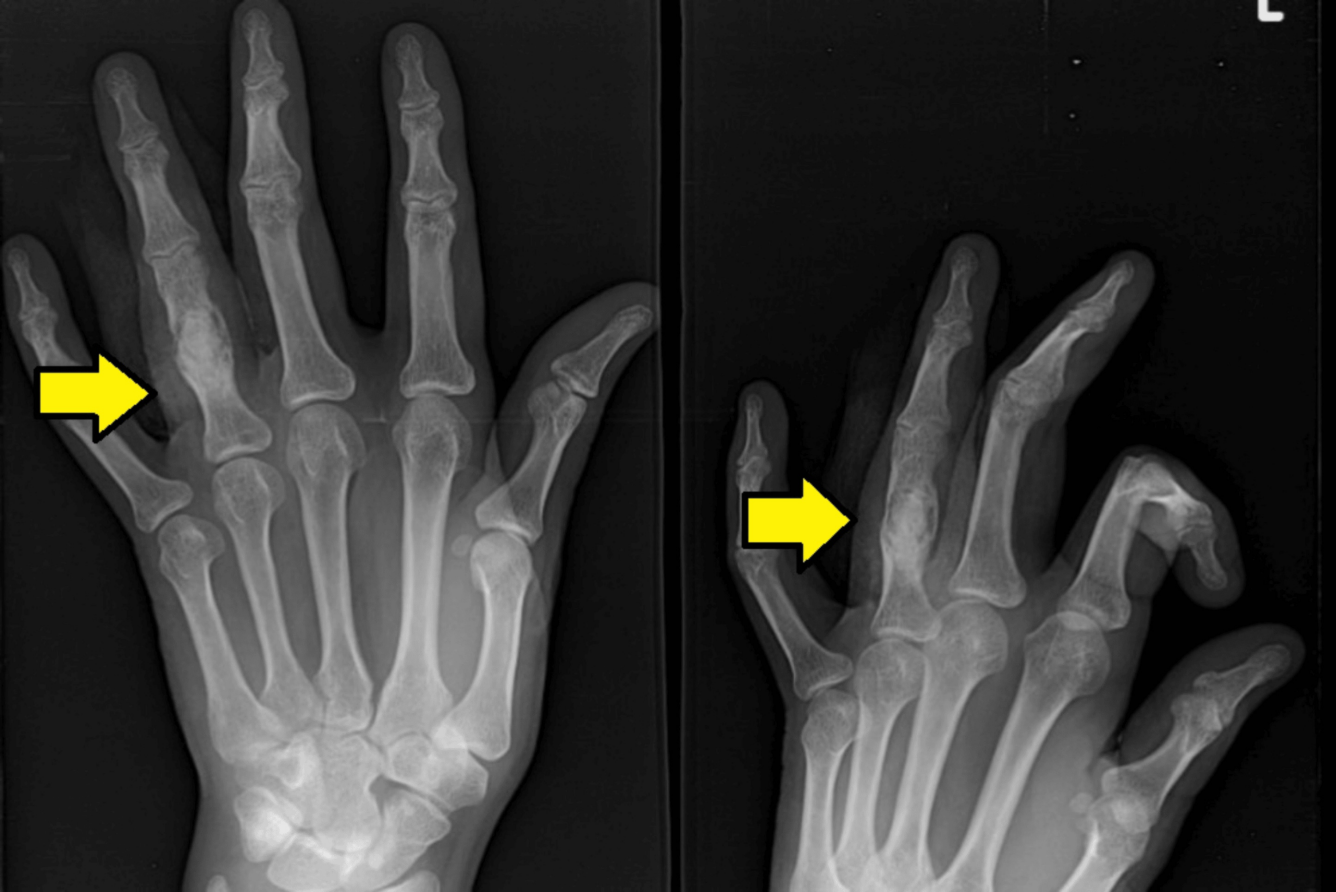
X-ray of the left hand showing sclerotic margins and a central diaphyseal lytic lesion in the proximal phalanx of the fourth finger (arrows)

A 28-year-old male presented with a three-month history of swelling on the proximal phalanx of the fourth finger. He had no complaints of fever or weight loss. Baseline investigations were performed and showed normal results. Seven years earlier, he had undergone k-wire fixation and bone grafting for the treatment of an enchondroma in the same phalanx. Recent X-ray imaging of the left hand revealed sclerotic margins and a central diaphyseal lytic lesion (Figure 1).

Following this, bone curettage was performed, and the tissue was sent for histopathological examination. Grossly, we received a gray-white globular soft tissue measuring 1.5 × 1 × 1 cm. The cut surface revealed gray-white areas with a small nodule measuring 0.5 × 0.5 cm. Additionally, we received multiple gray-white soft tissue fragments totaling 1 × 0.5 cm.

**Figure 2:**
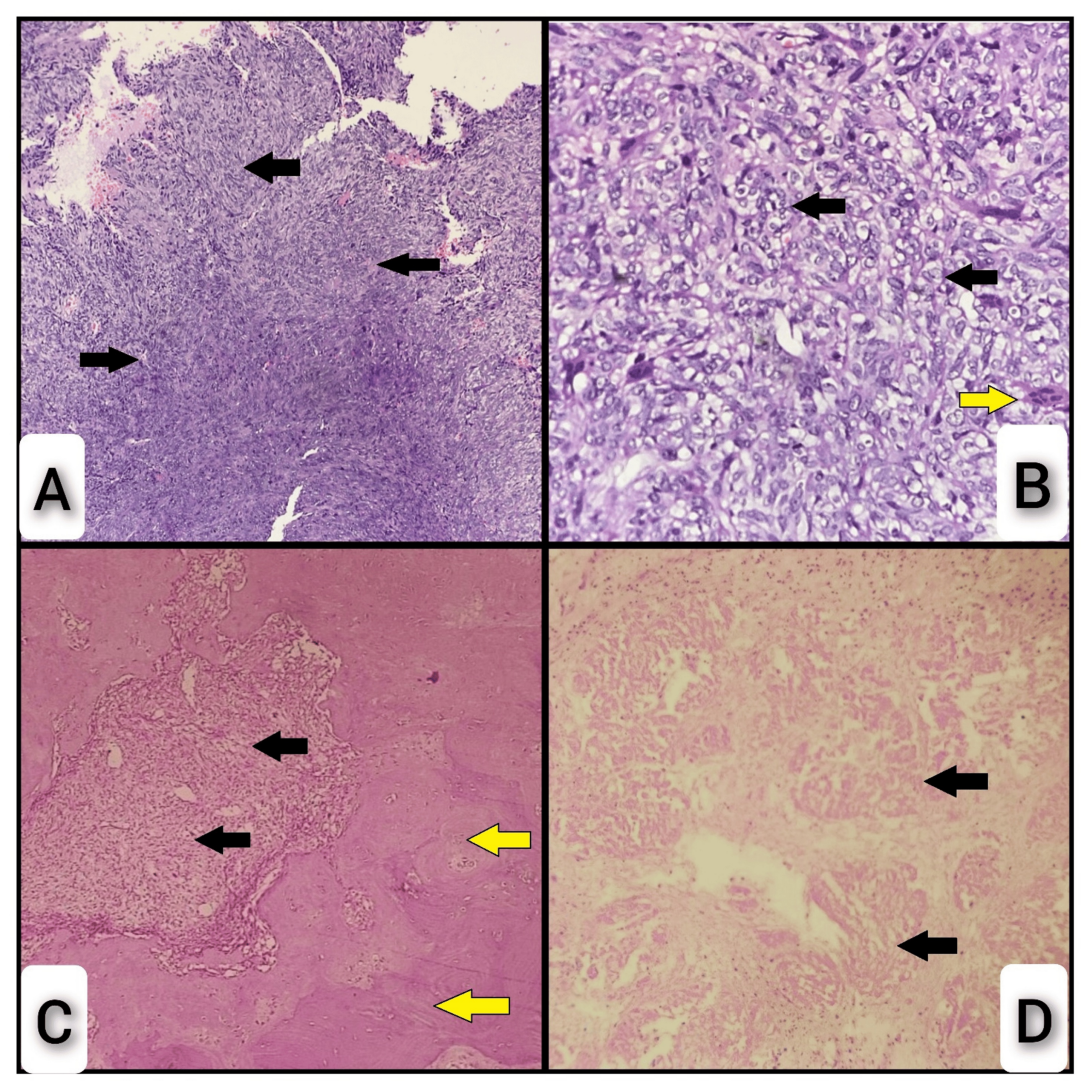
Microscopy (A) Malignant cellular neoplasm displaying sheets of oval to spindle-shaped cells (arrows) (H&E) – 4X. (B) Sheets of atypical oval to spindle cells with scant to moderate eosinophilic cytoplasm and moderately pleomorphic vesicular nuclei (black arrows), with osteoclast-type multinucleated giant cells scattered among the tumor cells (yellow arrow) (H&E) – 40X. (C) Tumor cells with vacuolated clear cytoplasm (black arrows), surrounded by chondroid material (yellow arrows) (H&E) – 4X. (D) Sheets of tumor cells with necrotic areas (arrows) (H&E) – 20X.

Microscopically, multiple sections revealed a cellular neoplasm composed of sheets of oval to spindle-shaped cells with scant to moderate eosinophilic cytoplasm and moderately pleomorphic vesicular nuclei, some of which had prominent nucleoli. Many areas exhibited dispersed osteoclast-type multinucleated giant cells amidst the tumor cells (Figure 2a and 2b). A few regions showed tumor cells with vacuolated clear cytoplasm surrounded by chondroid material (Figure 2c). Scattered mitotic figures (one to two per high-power field) were noted, along with areas of necrosis and hyalinization (Figure 2d).

**Figure 3:**
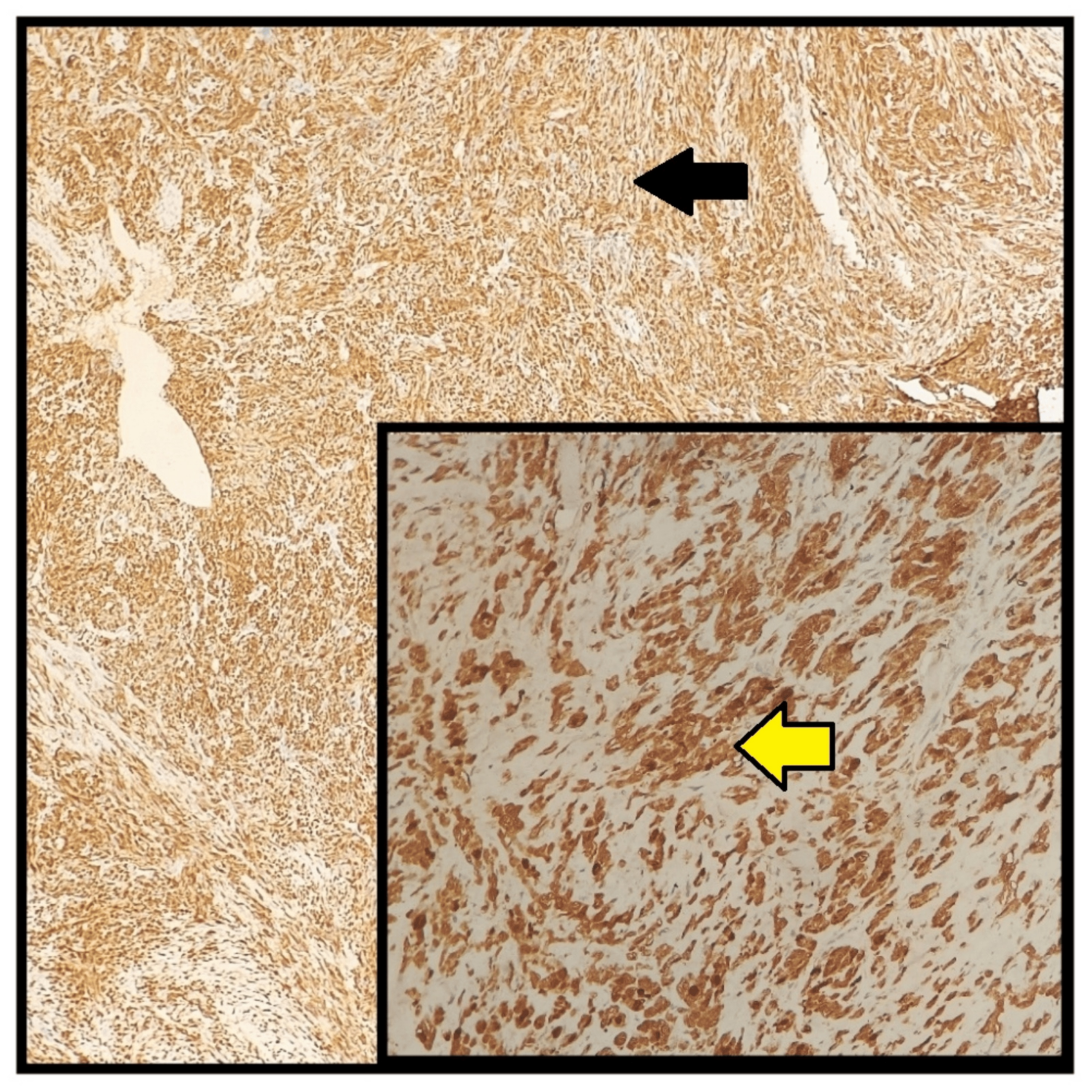
S100 immunohistochemistry Atypical cells showing 80% cytoplasmic positivity for S-100 (arrows), with an inset displaying a higher magnification image.

**Figure 4:**
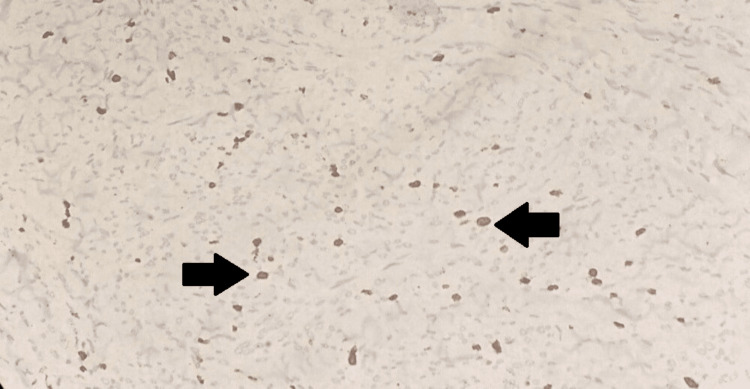
Ki 67 immunohistochemistry Atypical cells showing 10% nuclear positivity for the cell proliferation marker Ki-67 antigen (arrows).

Hence, a probable diagnosis of DDCS was made. An immunohistochemical analysis was performed using S-100 and Ki-67 as markers. The analysis revealed that 80% of the tumor cells exhibited significant cytoplasmic positivity for S-100 (Figure 3). Additionally, 10% of the tumor cells were positive for the cell proliferation marker Ki-67 (Figure 4).

The diagnosis was confirmed, and the patient underwent a ray amputation of the left fourth finger. Gross examination revealed that the tumor involved the metaphysis and diaphysis, extending into the soft tissue. The exact size could not be determined due to previous curettage. Microscopically, according to the American Joint Committee on Cancer 8th edition, the tumor was classified as pT1 (tumor ≤8 cm in greatest dimension). The N category was NX, as regional nodes could not be assessed. All margins were negative for the tumor. Consequently, a final diagnosis of high-grade DDCS, pT1 pNX, was made. The patient completed two cycles of chemotherapy (doxorubicin 75 mg, cisplatin 120 mg, ifosfamide 10 g, and methotrexate 8 g) and is currently in a follow-up period of one year.

## Discussion

In contrast to conventional CSs or non-cartilaginous sarcomas, DDCSs are extremely aggressive neoplasms with a high mortality rate. The prognosis is poor, with a five-year disease-free survival rate ranging from 10% to 24% [3]. CSs affecting the hand and foot are infrequent, and DDCS occurring in the distal extremities is even rarer [1].

A distinctive feature of DDCS is its bimorphic histological composition. It comprises a well-differentiated cartilaginous component, often resembling an enchondroma or low-grade CS, and an abrupt transition to a high-grade sarcoma lacking a cartilaginous component [1]. The dedifferentiated component can display a range of characteristics, including malignant fibrous histiocytoma (MFH), osteosarcoma, fibrosarcoma, angiosarcoma, rhabdomyosarcoma, leiomyosarcoma, and spindle cell sarcoma. One study found that patients with MFH have a notably lower median survival time (five months) compared to those with osteosarcoma (17 months) and spindle cell sarcoma (eight months) [3]. Conversely, patients with myofibroblastic sarcoma have been disease-free for approximately 60 months, suggesting that histological subtypes may impact survival [3].

DDCS typically presents with symptoms such as pain, localized swelling, restricted mobility, soft tissue masses, and pathological fractures. It most commonly affects long bones like the tibia, humerus, and femur, particularly in their proximal regions, where tumor cells are often distributed within the metaphysis and diaphysis. Tumor size greater than 5 cm and localization in the axial skeleton are strong indicators of malignancy. MRI reveals varying degrees of soft tissue and intraosseous involvement [6,7].

“Synchronous (primary) DDCS” refers to DDCS occurring at the site of a previously low-grade CS or enchondroma, while “metachronous (secondary) DDCS” refers to recurrence following the removal of a chondrogenic tumor. The removal of tumors with clear margins is associated with a higher chance of recovery. Although obtaining wide margins has been a good prognostic indicator for non-DDCS, its benefit in dedifferentiated cases remains uncertain [8].

IDH1/2 mutations are commonly seen in benign and malignant central and periosteal cartilage cancers, but they are not reliable markers for malignancy. However, since chondroblastic osteosarcomas typically lack IDH mutations, these can help distinguish them from high-grade or DDCS [9]. Comparing mutant IDH CSs to IDH non-mutant CSs, the latter exhibit higher metabolic activity [10]. Given the potential for a minimally dedifferentiated component, pathologists must carefully examine cartilaginous tumors to identify any aberrant areas. Due to biopsy constraints, obtaining both cartilage and dedifferentiated components simultaneously for the diagnosis of primary DDCS is challenging.

Overall, DDCS has a dismal prognosis, with widespread metastases being common, and most patients die within two years. A CT-guided puncture can significantly improve diagnostic accuracy, and combining a biopsy with a thorough patient history can facilitate the diagnosis of secondary DDCS.

## Conclusions

We herein reported a detailed case of DDCS located in the proximal phalanx of the fourth finger. Due to its rarity, DDCS must be accurately diagnosed and differentiated from other primary malignant sarcomas. Given its aggressive clinical behavior, high recurrence rate, and poor prognosis, early diagnosis of DDCS is critical. Although the grade of the cartilaginous component and local recurrence were not linked to survival, the histological subtype of dedifferentiation might influence outcomes. Identifying dedifferentiation before surgery is crucial for defining safe resection margins and improving the effectiveness of adjuvant therapy. We emphasize that a thorough approach, integrating clinical, radiological, and pathological data, is essential for diagnosing DDCS. A comprehensive evaluation of imaging characteristics and clinical history is necessary when assessing tissue specimens.
